# Effect of surface topography and wettability on shear bond strength of Y-TZP ceramic

**DOI:** 10.1038/s41598-023-45510-8

**Published:** 2023-10-25

**Authors:** Suriyakul Wongsue, Ornnicha Thanatvarakorn, Taweesak Prasansuttiporn, Piyarat Nimmanpipug, Thanapat Sastraruji, Keiichi Hosaka, Richard M. Foxton, Masatoshi Nakajima

**Affiliations:** 1 Dental Section, Buddhasothorn Hospital, Na Muang, Muang, 24000 Chachoengsao Thailand; 2 Faculty of Dentistry, Bangkokthonburi University, 16/10 Leab Klong Taweewatana Rd., Taweewatana, 10170 Bangkok Thailand; 3 Department of Restorative Dentistry and Periodontology, Faculty of Dentistry, Chiang Mai University, Suthep, Muang Chiang Mai, 50200 Chiang Mai Thailand; 4 Department of Chemistry, Faculty of Science, Chiang Mai University, Suthep, Muang Chiang Mai, 50200 Chiang Mai Thailand; 5 Dental Research Center, Faculty of Dentistry, Chiang Mai University, Suthep, Muang Chiang Mai, 50200 Chiang Mai Thailand; 6 Department of Regenerative Dental Medicine, Graduate School of Biomedical Sciences, Tokushima University, 3-18-15, Kuramoto-cho, Tokushima, 770-8504 Japan; 7 King’s College London Dental Institute, King’s College London, Floor 25, London Bridge, London, SE1-9RT UK; 8 Department of Cariology and Operative Dentistry, Graduate School of Medical and Dental Sciences, Tokyo Medical and Dental University, 1-5-45, Yushima, Bunkyo-ku, Tokyo 113-8549 Japan

**Keywords:** Chemistry, Materials science

## Abstract

Zirconia ceramics have been widely used as dental restorations due to their esthetic appearance and high flexural strength. The bonding of zirconia with resin cement should rely on both mechanical and chemical bonds. This study was performed to investigate the effect of zirconia surface topography and its wettability after surface pretreatments on the microshear bond strength (μSBS) of a resin cement. Zirconia slabs were prepared and randomly divided into 5 groups based on the surface treatment as follows: no treatment (control), air abrasion (AB), etching with hydrofluoric acid (F), the mixture of hydrofluoric acid and nitric acid (FN), or the mixture of hydrochloric acid and nitric acid (CN) for 10 min. The specimens were subjected to investigation of surface roughness characteristics [average roughness (Ra), peak-to-valley average distance (Rpv), skewness (Rsk), and kurtosis (Rku)] using atomic force microscopy (AFM) and measurements of surface contact angle (*θ*_c_) and μSBS of a resin cement. In addition, the area % of the nanoscale surface irregularity (nSI%) was calculated from the AFM images. The effects of nSI%, Ra and *θ*_c_ on the μSBS were analyzed by multiple linear regression analysis (*p* < 0.05). Multiple regression analysis revealed that the nSI% was the most predominant factor for the μSBS (*p* < 0.001). A surface with larger nSI%, higher Ra and relatively lower *θ*_c_ was essential for establishing a reliable resin-zirconia bond.

## Introduction

Yttria-stabilized tetragonal zirconia polycrystal (Y-TZP) ceramics have been widely used in restorative dentistry due to their esthetic appearance and high flexural strength^1^. However, the bonding of zirconia with resin cement is still considered problematic. Unlike glass ceramics, these ceramics cannot be etched with conventional 5–9% hydrofluoric acid (HF) to create the surface roughness necessary for mechanical interlocking. Thus, the standard protocol is airborne-particle abrasion for mechanically roughening the surface^2,3^, accompanied by the application of 10-methacryloyloxydecyl dihydrogen phosphate (10-MDP) monomer to facilitate chemical bonding^4^. This protocol has been reported to be successful in achieving optimal zirconia-resin bond strength and bond durability^5–7^.

However, air abrasion was reported to produce sharp-edged irregularities, which might cause initiation of crack propagation in the material^8^. Moreover, a too deep and narrow rough surface of adherend materials would reduce the penetration of adhesives or resin cements^9,10^, leading to prevention of the optimal formation of mechanical interlocking and/or chemical bonding. Meanwhile, it was speculated that a shallow and wide-open rough surface would be more favorable in allowing complete filling of resin cement^11^.

Concurrently, HF at a high concentration was introduced to etch the zirconia and provide roughness on the zirconia surface, manifested as micro- and nano-porosity^11,12^. Although the formation of nano-porosity did not drastically increase the average surface roughness (Ra) value, compared to air abrasion, its presence was found to increase surface wettability and improve the resin-zirconia bond strength^11^.

Regarding roughness measurement, zirconia bonding studies generally report treated zirconia surface topography by the Ra value, which evaluates the surface irregularities in the vertical direction. It should be noted that Ra only measures the height average deviation from the mean line, where the slopes, sizes, geometries of peaks and valleys, or nanoscale irregularity on the microrough surface cannot be exactly detailed^13^. Therefore, other surface roughness parameters, such as the average distance from the tips of peaks to valleys (Rpv), the height distribution and tip geometry of peaks and valleys evaluated by skewness (Rsk) and kurtosis (Rku), and the presence of nanoscale surface irregularity (nSI), should be investigated because they might be able to affect resin penetration and, consequently, the resin-zirconia bond strength.

Thus, the objective of this study was to investigate the effect of surface topography, determined by Ra, Rpv, Rsk, and Rku, the surface wettability, and the nSI of pretreated zirconia ceramics on the microshear bond strengths (μSBS) of a resin cement to zirconia. The null hypothesis tested was that the surface topography and wettability do not affect resin-zirconia bond strengths.

## Methods

### Zirconia specimen preparation and surface treatment

Sixty zirconia slabs were prepared from partially-sintered zirconia milling blanks (BruxZir^®^ Shaded; Glidewell Laboratories, CA, USA) by using a low-speed diamond saw (IsoMet™ low speed cutter; Buehler, IL, USA), and the top and bottom surfaces of slabs were polished under water running with abrasive SiC papers (600, 800, 1000, 1200, and 2000 grits, respectively) using a grinding machine (MoPao™ 160E, Laizhou Weiyi, Shandong, China). Then, all the specimens were fully sintered into the final dimensions of 10 × 10 × 2 mm following the manufacturer’s instructions and randomly assigned into 5 groups (n = 12) as follows: Control: no surface treatment, Group AB: air abrasion with 50 μm alumina particles under 2 bars at 10 mm from the specimen top surface for 10 s followed by 10 min ultrasonic cleaning in deionized water, Group F: etching with 48% HF, Group FN: etching with 48% HF mixed with 68% nitric acid (HNO_3_) at a 1:1 ratio by volume, and Group CN: etching with 37% hydrochloric acid (HCl) mixed with 68% HNO_3_ at a 4:1 ratio by volume. In the acid etching groups, 50 µL of designated acid was dropped and thus wetted the zirconia surface for 10 min, followed by rinsing with deionized water for 1 min. The chemical agents used are listed in Table 1. To eliminate the variation in surface chemistry in each treatment, carbon was intentionally allowed to absorb on the specimen surfaces by storing the specimens in an empty closed container at room temperature for 21 days before the following experiments.

**Table 1:** Materials used in this study.

Agents	Manufacturer	Composition	Application
HF (Emsure® Hydrofluoric acid)	Merck, Darmstadt, Hesse, Germany [B1004344747]	48% w/w HF	–
HNO_3_ (Gammaco™ Nitric acid)	Gammaco, Bang Kruai, Nonthaburi, Thailand [3095045]	68% w/w HNO_3_	–
HCl (Emsure® Hydrochloric acid)	Merck, Darmstadt, Hesse, Germany [K45311117505]	37% w/w HCl	–
Clearfil™ Ceramic Primer Plus	Kuraray Noritake Inc., Okayama, Japan [AU0049]	3-trimethoxysilylpropyl methacrylate, 10-MDP, Ethanol	Apply primer on zirconia surface and allow it to react for 10 s, then dry with mild air flow
Panavia™ V5; Dual cure mode, Clear color	Kuraray Noritake Inc., Okayama, Japan [7S0027]	Bis-GMA, TEGDMA, Hydrophobic aromatic dimethacrylate, hydrophilic aliphatic dimethacrylate, initiators, accelerators, silanated barium glass filler, silanated fluoroaluminosilicate glass filler, colloidal silica, silanated aluminium oxide filler, dl-camphorquinone, pigments	1. Attach a mixing tip to the syringe 2. Load mixed paste into polyethylene mold placed over zirconia surface 3. Remove excess cement, and light-cure for 20 s

*HF* Hydrofluoric acid, *HNO*_*3*_ Nitric acid, *HCl* Hydrochloric acid, *10-MDP* 10-Methacryloyloxydecyl dihydrogen phosphate, *Bis-GMA* 2,2-Bis[4-(2-hydroxy-3-methacryloyloxypropoxy)-phenyl) propane, *TEGDMA* Triethyleneglycol dimethacrylate.

### Surface topography assessment using atomic force microscopy (AFM)

#### Surface roughness characteristics measurement

All the specimens were investigated for surface roughness characteristics using AFM (PARK XE7, Park Systems, Gyeonggi, South Korea) in noncontact mode. The parameters Ra, Rpv, Rsk, and Rku were calculated using analysis software (XEI 4.3, Park Systems, Gyeonggi, South Korea). The Ra, Rpv, Rsk, and Rku data were analyzed statistically with one-way ANOVA and Tukey’s test (*p* < 0.05).

**Figure 1:**
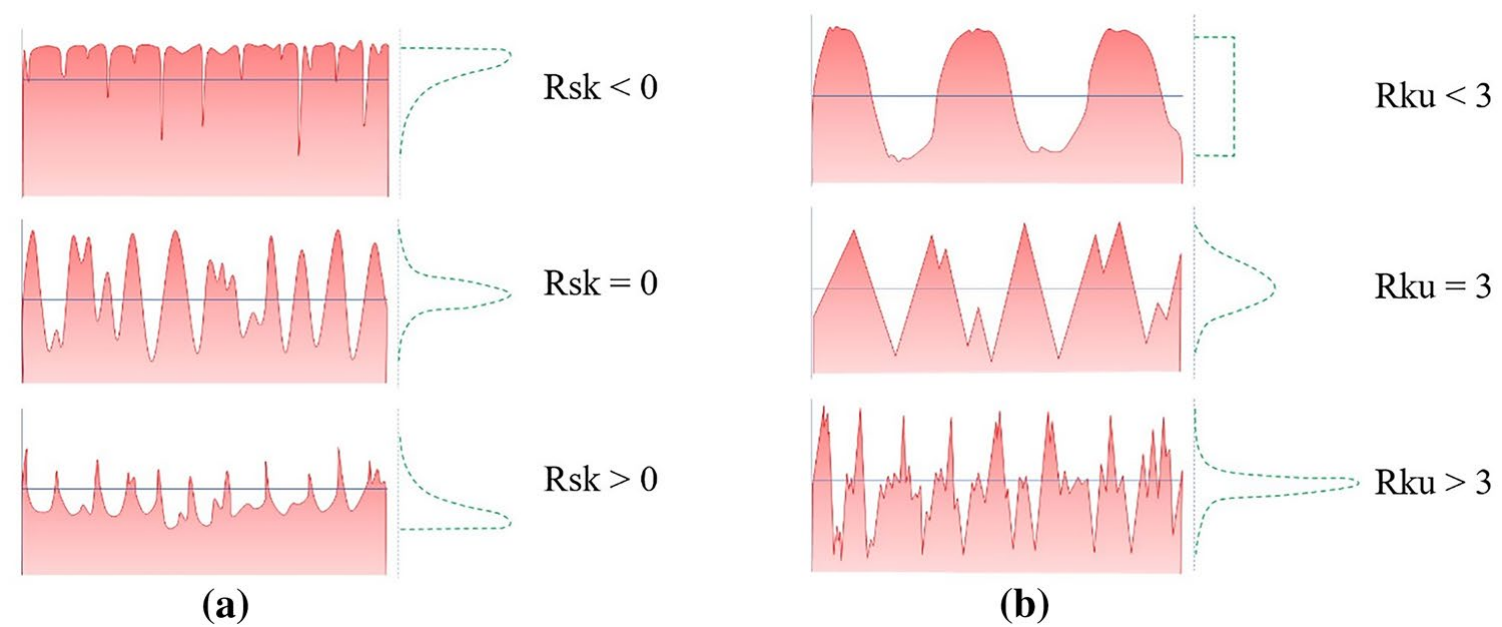
Schematic illustrations demonstrate the meaning of parameters Rsk (**a**) and Rku (**b**).

Rsk is a measure of the asymmetry of the profile about the mean line calculated over the evaluation length. Profiles with predominant deep valleys have negative skewness, whereas those with several high peaks show positive values. Rku is a measure of the peakedness of the profile about the mean line calculated over the evaluation length. It describes the sharpness of the probability density of the profile, of which Rku > 3 indicates sharp peaks and valleys, and Rku < 3 refers to relatively flat peaks and valleys. If Rku = 3, then the curve is Gaussian (Fig. 1)^14^.

#### nSI assessment

**Figure 2:**
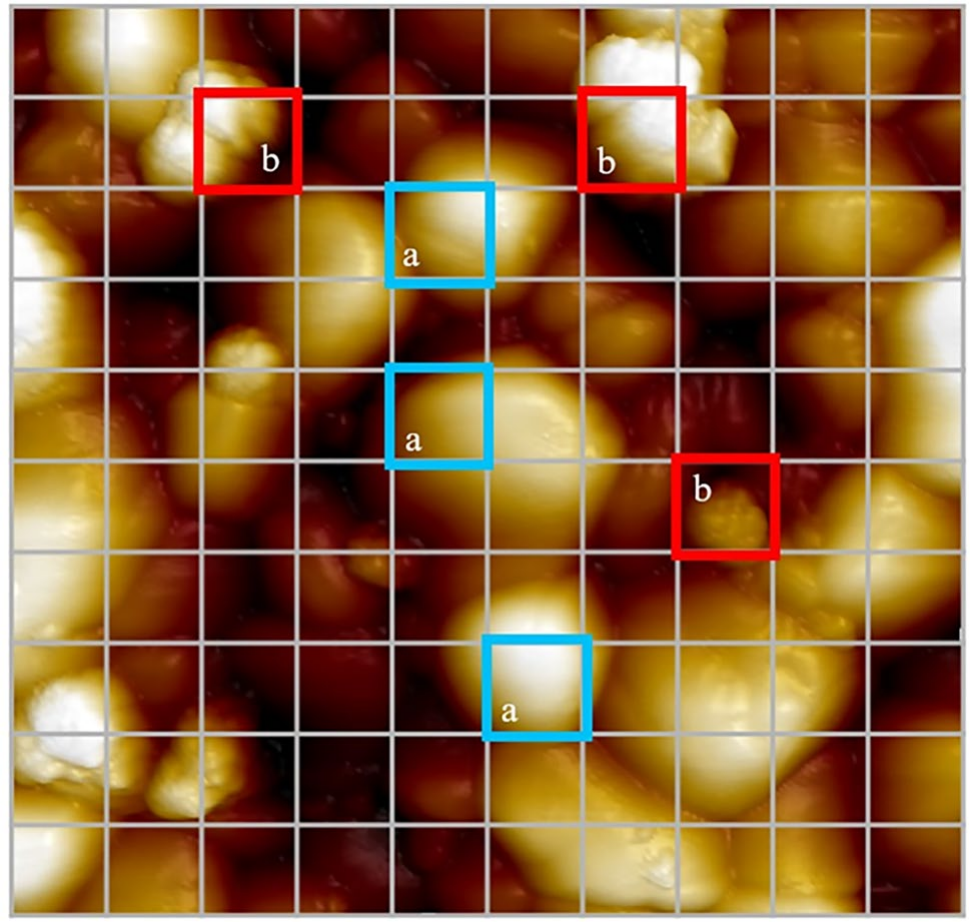
The representative 5 × 5 µm zirconia surface in a 2D image, showing the counting from a hundred-square grid. Blue squares (**a**) show smooth grain surface grids, and red squares (**b**) show nanoscale surface irregularity (nSI) grids.

To observe nanoscale surface topography, a 5 × 5 µm-AFM image with 256 × 256 pixels was taken with a scan rate of 0.5 Hz to assess nSI on the treated zirconia surface. The nSI area was quantitatively defined as a percentage of the whole surface (nSI%) by manual counting using a simple hundred-square grid from a 2D image. If a clearly smooth grain surface presented more than 50% of the square grid, it was counted as a smooth grain surface grid (Fig. 2a). Meanwhile, if the rough or irregular grain surface presented more than 50% of the square grid, it was counted as an nSI grid (Fig. 2b).

Twelve images from each group were measured by 2 observers, 2 times with a 1-week interval. A validation of the method used in this study was performed by both intraobserver and interobserver reliability analysis using the intraclass correlation coefficient (ICC) test, whose correlation ratios were 0.988 and 0.972, respectively (*p* < 0.05). The nSI% was analyzed statistically with one-way ANOVA and Tukey’s test (*p* < 0.05).

### Surface contact angle ($\it \uptheta_{c}$) assessment

All the treated specimens were used for contact angle measurement by the sessile drop technique. One microliter of deionized water was dropped on the specimen surface and left for 1 min prior to the measurement using a goniometer (CMU-PHYS™ Goniometer, CMU-PHYSnanolabs, Chiang Mai, Thailand). The *θ*_*c*_ data were analyzed statistically with one-way ANOVA and Tukey’s test (*p* < 0.05).

### μSBS test

After the investigations, all the specimen surfaces were applied with a 10-MDP containing primer (Clearfil™ Ceramic Primer Plus, Kuraray Noritake Inc., Okayama, Japan). After placing polyethelene tubes (Tygon^®^, Norton Performance Plastic, OH, USA) with an internal diameter of 0.8 mm and a height of 0.5 mm on the zirconia surface, resin cement (Panavia™ V5, Kuraray Noritake Inc., Okayama, Japan) was subsequently injected into the tube and light cured for 20 s from the top surface using a light-curing unit (Bluephase^®^ N, Ivoclar Vivadent, Schaan, Liechtenstein). All specimens were stored in 37 °C distilled water for 24 h. After carefully removing the tubes, the specimens were subjected to μSBS testing using a universal testing machine (Instron® 5566, Illinois Tool Works, MA, USA) using the wire-loop technique at a crosshead speed of 0.5 mm/min. The materials used are listed in Table 1. The μSBS data were analyzed statistically with one-way ANOVA and Tukey’s test (*p* < 0.05).

### Failure mode analysis

After the μSBS test, failure mode analysis was carried out using a stereoscopic microscope (Olympus™ SZX7, Olympus, Tokyo, Japan) at 56 × magnification. Failure modes were categorized into 3 types as follows^15^: *adhesive failure* (80–100% of the failure occurred at the interface between resin cement and zirconia), *cohesive failure* in resin cement or zirconia (80–100% of the failure occurred within resin cement or zirconia), and *mixed failure* (mixed with adhesive and cohesive failure patterns in the same specimen). The failure mode data were analyzed statistically with the chi-square test (*p* < 0.05).

### Multiple linear regression analysis

To investigate the effect of nSI%, Ra and *θ*_*c*_ on μSBS, multiple regression analysis was performed by using IBM SPSS Statistics version 27.0 (IBM, Chicago, IL, USA) (*p* < 0.05). Model fit was assessed using the coefficient of determination (R squared).

## Results

### Surface topography assessment using AFM

The means of Ra, Rpv, Rsk, Rku and their standard deviations are presented in Table 2. All the surface treatments exhibited significant increases in Ra and Rpv values compared with the control group (*p* < 0.05). The AB and F groups exhibited the highest Ra and Rpv values among the treatments. The Rsk values were negative in the control and CN groups, whereas they were near zero in the AB group and positive in the F and FN groups. The Rku value of the control group was more than 3. All the surface treatments caused an insignificant change in Rku compared with the control group (*p* > 0.05). The only significant difference in Rku was found between the CN group (highest Rku, more than 3) and the F group (lowest Rku, less than 3) (*p* < 0.05).

**Table 2:** Means and standard deviations of surface roughness parameters (Ra, Rpv, Rsk, Rku), area percentage of nanoscale grain-surface irregularity (nSI%), surface contact angle (*θ*_*c*_) and microshear bond strengths (μSBS) of each group (*n* = 12).

Surface treatments	Surface roughness parameters				nSI%	*θ*_*c*_ (°)	µSBS (MPa)
	Ra (nm)	Rpv (nm)	Rsk	Rku			
Control	47.17 ± 5.44^a^	448.62 ± 78.38^a^	− 0.23 ± 0.28^a^	3.22 ± 0.62^ab^	16.33 ± 10.36^a^	83.33 ± 3.22^d^	8.59 ± 1.22^a^
AB	109.39 ± 7.82^c^	974.56 ± 224.42^c^	0.00 ± 0.21^abc^	3.14 ± 0.51^ab^	81.00 ± 7.07^c^	73.18 ± 6.06^c^	19.37 ± 2.58^c^
F	104.16 ± 7.55^c^	893.74 ± 148.61^c^	0.09 ± 0.21^bc^	2.87 ± 0.32^a^	92.17 ± 6.59^d^	63.22 ± 5.71^b^	22.21 ± 2.35^d^
FN	73.16 ± 7.59^b^	706.78 ± 140.88^b^	0.17 ± 0.27^c^	3.02 ± 0.48^ab^	91.25 ± 8.29^d^	53.19 ± 7.59^a^	19.26 ± 2.00^c^
CN	69.13 ± 7.55^b^	648.91 ± 142.96^b^	− 0.21 ± 0.39^ab^	3.53 ± 0.69^b^	64.33 ± 8.92^b^	72.76 ± 5.77^c^	13.78 ± 1.80^b^

Different superscript letters indicate statistically significant differences between surface treatments (*p* < 0.05).

**Figure 3:**
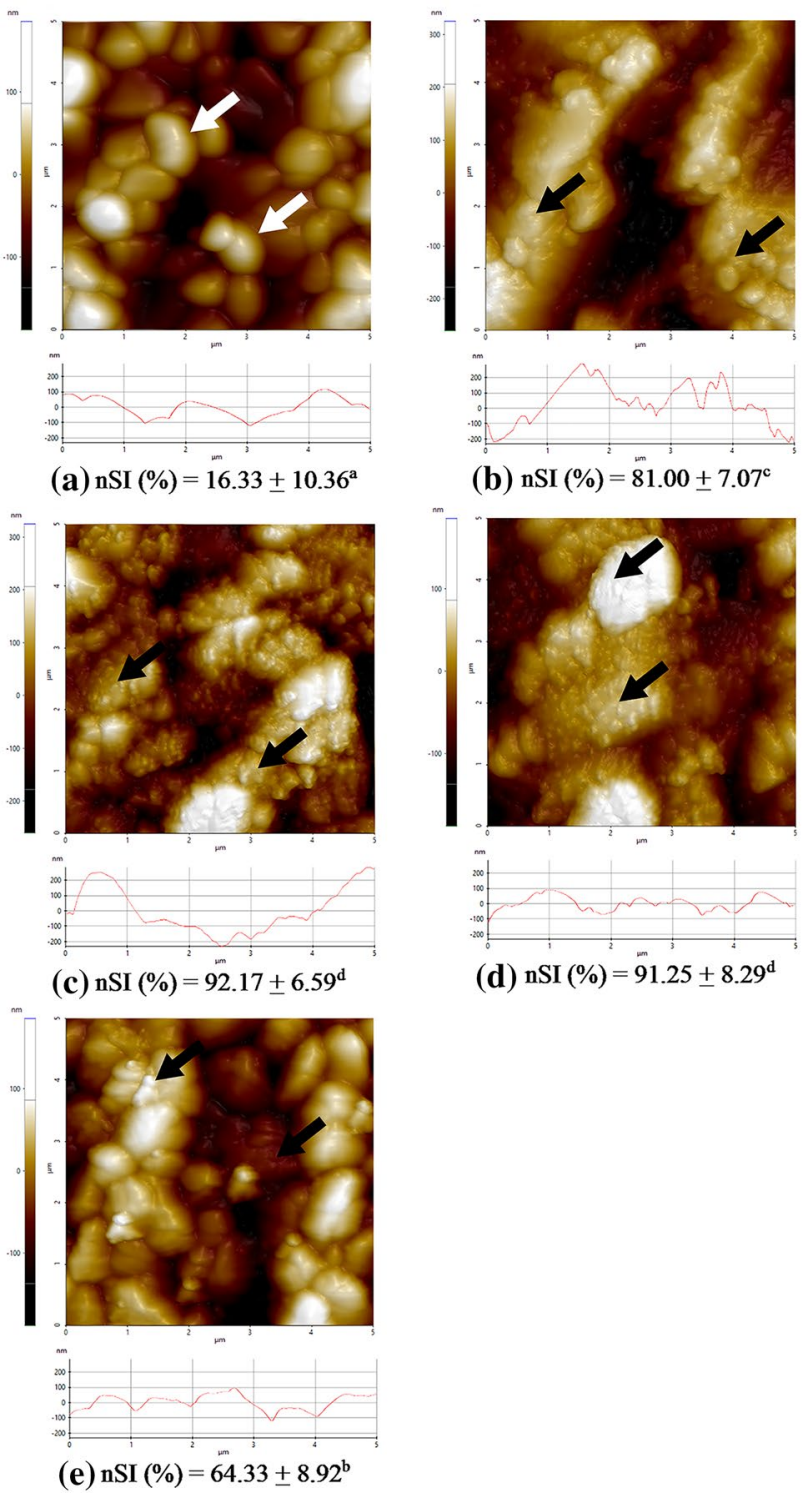
Representative AFM images (upper) and line-scan profiles (lower) of the control (**a**), AB (**b**), F (**c**), FN (**d**), and CN (**e**) groups. Grain boundaries (white arrow) were clearly observed in the control group, whereas surface irregularities on grains (black arrows) were observed in all treated groups.

Representative AFM images exhibiting the surface topography of nontreated and surface-treated zirconia are shown in Fig. 3. Smooth surface grains and grain boundaries were clearly observed in the control group. On the other hand, the F and FN groups clearly exhibited nSI on grains with unclear grain boundaries. In the AB group, nSI with unclear grain boundaries was observed in the lesser amount. For the CN group, small protrusions were observed on the smooth surface grains with traceable boundaries.

The means and standard deviations of nSI% are shown in Table 2. All the treatments significantly increased nSI% compared with the control group (*p* < 0.05), and the F and FN groups showed significantly higher nSI% than the AB and CN groups (*p* < 0.05).

### $\it \theta_{c}$ assessment

The mean *θ*_*c*_ and standard deviations are shown in Table 2. All the surface treatments significantly decreased *θ*_*c*_ compared with the control group (*p* < 0.05), in which the FN group exhibited significantly lower *θ*_*c*_ among the treatment groups (*p* < 0.05).

### μSBS test

The means and standard deviations of μSBS in each group are shown in Table 2. All the treatments significantly increased μSBS compared with the control group (*p* < 0.05), and the F group exhibited significantly higher μSBS among the treatment groups (*p* < 0.05).

### Failure mode analysis

**Figure 4:**
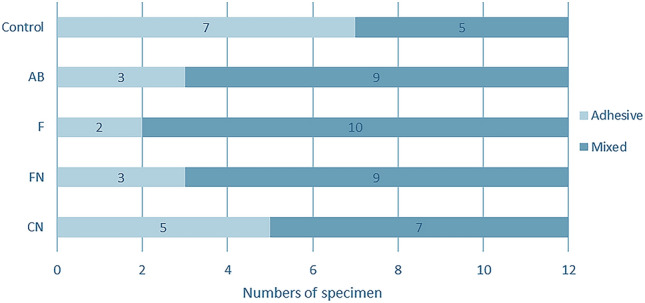
Frequency of failure modes after µSBS testing in each group (*n* = 12). The numbers in each bar are the numbers of specimens in each failure mode. There were significant differences in the failure mode distribution among the groups (*p* < 0.05). The majority of the failure mode of the control was adhesive failure, whereas it was mixed failure in the AB, F, FN, and CN groups.

The failure mode distribution is displayed in Fig. 4. The results of the chi-square test revealed that there were significant differences in the failure mode distribution among the groups (*p* < 0.05). The majority of the failure mode of the control was adhesive failure, whereas it was mixed failure in the AB, F, FN, and CN groups.

### Multiple linear regression analysis

The regression model with nSI%, Ra and *θ*_*c*_ was significantly fitted to *μ*SBS [*μ*SBS = 9.487 + 0.089(nSI%) + 0.080(Ra) – 0.079(*θ*_*c*_)] (*p* = 0.003; R^2^ = 0.831), in which nSI%, Ra, and *θ*_*c*_ were statistically significant predictors (*p* < 0.001, *p* < 0.001 and *p* = 0.035, respectively). The nSI% was the most predominant factor for resin-zirconia bond strength (standardized coefficient beta = 0.499) (Table 3).

**Table 3:** Multiple linear regression analysis.

Model	Sum of squares	df	Mean square	F	Sig.
ANOVA					
Regression	1371.053	3	457.018	91.479	< 0.001
Residual	279.769	56	4.996		
Total	1650.823	59			

Model	Unstandardized coefficients		Standardized coefficients Beta	Sig.	Collinearity statistics VIF
	B	Std. Error			
Constant	9.487	3.090		0.003	
nSI%	0.089	0.020	0.499	< 0.001	3.960
Ra	0.080	0.018	0.367	< 0.001	2.332
*θ*_*c*_	− 0.079	0.036	− 0.174	0.035	2.146

R^2^ = 0.831.

## Discussion

The results of this study revealed that the air-abrasive and acid-etching treatments used in this study caused variations in the surface topography (Ra, Rpv, Rsk, Rku and nSI%) of the zirconia surface and affected the *θ*_*c*_ and bonding performance of the resin cement. Additionally, nSI%, Ra and *θ*_*c*_ significantly affected μSBS between resin and zirconia. Thus, the null hypothesis was rejected.

AFM was employed in this study since it is an essential tool for characterizing surface topography, particularly on the nanoscale, providing a direct view of surface features at high resolution without surface coating being required^16^.

With the aim of creating zirconia surfaces with various micro- and nanoscale irregularities, the treatments of airborne particle abrasion or acid etching with HF and its mixture with HNO_3_, including a mixture of HCl with HNO_3,_ were used. Air-pressurized abrasion can roughen the surface with large surface defects and mechanically damage the zirconia grains in the top 1- to 1.5-µm zone^17^. HF etching using a high concentration can corrode both intergranular and intragranular parts of the zirconia surface, being slightly faster at the grain boundaries^18^, leading to a size reduction and dislodgement of zirconia grains^12^. These two methods increased Ra and Rpv to the highest values, whereas the mixture of HF with HNO_3_ (FN group) and the mixture of HCl with HNO_3_ (CN group) caused the lesser increase in Ra and Rpv. The results of this study were consistent with previous studies, which reported that the mixture of HF with HNO_3_ was capable of roughening a zirconia surface^2,19,20^ but to a lesser extent than HF etching alone^21^. It was speculated that adding HNO_3_ reduced the penetration of the mixture into grain boundaries, no longer dislodging the deeper-part grains. Last, the mixture of HCl with HNO_3_ barely etched the zirconia substrate, although its strong oxidative effect^22^ and its ability to readily dissolve common metallic oxides and hydroxides^23^ have been addressed.

In adhesive dentistry, adequate surface wetting of the adhesive on the substrate is the primary requirement to achieve good adhesion^24^. Good surface wetting, determined by a low contact angle, leads to intimate contact between adhesive and adherend and enhances mechanical interlocking^25,26^. Thus, surface wettability could be one of the parameters used to predict bonding efficacy. It has been documented that the contact angle is the parameter manifested from the substrate’s surface roughness and surface chemistry^27^. To eliminate the effect of chemical heterogeneity on the treated surfaces, each specimen was stored in a closed container for 21 days before contact angle measurement. This aging condition possibly exerted airborne hydrocarbon adsorption, which subsequently increased the contact angle^27,28^. Although this method overestimated the values of measured *θ*_*c*_, it aided the surface chemistry of all treated groups to be homogenized, and hence, the wettability derived from the effect of surface topography could be predominant^29^.

The Wenzel theory states that an increase in surface roughness could decrease the contact angle for a hydrophilic surface^30,31^. However, several studies demonstrated that the contact angle decreases following increasing roughness to a certain extent, where no greater improvement in wetting is expected for highly rough surfaces^32,33^. It should be noted that the wettability was determined not only by the average surface roughness but also by the surface geometry in three dimensions, such as the distance between peaks compared to their height, of which the proper distance and height could facilitate the liquid to better wet the surface by forming a capillary action^32^. Excessively high peaks and symmetrically distributed high peaks and deep valleys (near-zero Rsk) conversely decrease wettability (increase *θ*_*c*_) due to trapped air in surface cavities^33,34^, whereas flat-shaped peaks and valleys (Rku < 3) are more favourable for enhancing surface wettability^35^. From the results of this study, the FN group exhibited the lowest *θ*_*c*_, which could be attributed to an optimal height and a predominance of peaks (positive Rsk) having a symmetric Gaussian shape (Rku = 3). On the other hand, both the AB and F groups created higher rough surfaces, which might be too high, with a more symmetric topography (near-zero Rsk). Thus, the wettability of the AB and F groups was hampered compared to that of the FN group. When compared to each other, the shape of peaks and valleys was relatively sharp in the AB group (Rku > 3) and flat in the F group (Rku < 3), leading to the better wettability of the F group than that of the AB group. Last, the *θ*_*c*_ values of the control and CN groups were the highest among the studied groups due to their low surface roughness and predominance of sharp valleys (negative Rsk and Rku > 3). Presumably, the moderately high roughness, with flat peaks (greater skewness and small kurtosis), might be preferable to enhance liquid wettability on the zirconia surface.

Interestingly, nSI was obviously observed on the grain surfaces of treated zirconia, especially in the F and FN groups. Since this nSI could not be quantified by the roughness parameters described above, the nSI% was evaluated manually by counting under the hundred-square grid method. It was speculated that a zirconia surface with a higher nSI% could increase the surface area for adhesion; thus, a higher bond strength could be expected. Accordingly, the F and FN groups, including the AB group, which had a high nSI%, demonstrated a higher µSBS than the groups with a lower nSI%.

Regarding the µSBS results, the highest µSBS was found in the F group, although its wettability was not the highest. The AB group had comparable µSBS to the FN group, despite its lower wettability and lower nSI% than that of FN. These results indicated that not only the wettability, defined by *θ*_*c*_, and the nSI%, but also the surface roughness (Ra), which was highest in the F and AB groups, could cooperatively contribute to improving the bond strength of resin cement to zirconia. This speculation was confirmed by the multiple regression analysis, revealing that nSI%, Ra and *θ*_*c*_ significantly affected the µSBS (*p* < 0.001, *p* < 0.001 and *p* = 0.035, respectively), in which nSI% was the most predominant factor contributing to the bonding to zirconia (standardized coefficient beta = 0.499), and Ra was a more significant predictor than *θ*_*c*_ (standardized coefficient beta = 0.367 and − 0.174, respectively) (Table 3). Therefore, the surface roughness would be an essential parameter for bonding with mechanical interlocking by resin tags to the zirconia surface, and the surface wettability would assist their optimal formation. In this analysis, the variance inflation factor (VIF) value of each predictor was less than 5 (Table 3), which indicates that the multicollinearity between the variables of nSI%, Ra and *θ*_*c*_ was small. Therefore, the nSI% would not be strongly associated with the wettability on the surface.

It is noteworthy that all the surface treatments could increase bond strengths compared with the control group. These findings are in agreement with other studies^21,36,37^, indicating that roughness is indispensable for the mechanical bond of resin-zirconia. Unlike the results from this study, a higher bond strength of air abrasion over HF treatment has been reported^21,38^. Additionally, some studies have demonstrated that the use of HF/HNO_3_ could increase bond strength to a larger degree than air abrasion^19,37^. These inconsistent results could be attributed to the differences in acid concentration, etching time, or resin cement types^19,39,40^. In particular, the 3-week waiting period prior to cementation in this study might affect the chemical bonds of each treatment to different degrees.

Within the methodology in the current study, there are some clinical limitations to be considered. First, it would be impractical to treat the intaglio surface of a crown and wait 3 weeks before proceeding with cementation. Second, the hazards of HF are well recognized^41^. Working with highly concentrated HF requires an isolated workspace with adequate ventilation, which is unfeasible in general clinical practice. Finally, the tetragonal-to-monoclinic (*t*–*m*) phase transformation, which is a critical consideration for the surface treatment of Y-TZP ceramics, was not evaluated in this study. Previous studies demonstrated that gentle air abrasion protocols, as used in this study, generally trigger *t*–*m* phase transformation^42–44^, whereas HF etching with conditions close to the current study was found to induce phase transformation to the lesser degree than air abrasion^45^. Nevertheless, such a small amount of monoclinic phase presented on the zirconia surfaces prior to the bonding procedure was found to not influence the resin bond strength^45–47^.

After aging, the increase in monoclinic contents^48^ and the decrease in bond strengths of resin-zirconia^45,49,50^ were widely reported. However, the study revealing the negative correlation between monoclinic contents and bond strengths after aging is scarce. On the other hand, some previous studies showed increased monoclinic contents without a significant drop in resin bonding^51,52^. It was concluded that the effect of resin hydrolysis is responsible for the decreased bond strength more than the phase transformation^21,52^. Moreover, it is still controversial whether the development of residual stresses at the interface following phase transformation may negatively affect surface adhesion^47,50^. Therefore, further study investigating the effect of *t*–*m* phase transformation on both immediate and long-term bond strengths is needed.

It could be concluded based on this in vitro study that appropriate surface topography and surface wettability are essential for establishing reliable resin-zirconia bond strength. Of all the factors evaluated, nSI% was the most predominant factor, whereas Ra and *θ*_*c*_ could concomitantly affect the bond strengths of resin cement to zirconia. The surface roughness properties Rsk and Rku are important parameters for facilitating surface wettability.

## Data Availability

The datasets generated during and/or analysed during the current study are available from the corresponding author on reasonable request.
